# Multilayer Genomic Characterization of a Shared Genetic Factor Linking Depression-Related Liability and Reduced Physical Function

**DOI:** 10.3390/genes17070813

**Published:** 2026-07-16

**Authors:** Wen Zeng, Xiupeng Yang, Yonggang Xu

**Affiliations:** Department of Hematology, Xiyuan Hospital, China Academy of Chinese Medical Sciences, Beijing 100091, China

**Keywords:** genomic SEM, shared genetic architecture, depression-related liability, reduced physical function, post-GWAS analysis, spatial transcriptomics

## Abstract

**Background:** Depression-related liability is frequently accompanied by reduced physical function, yet the shared genetic architecture linking mood-related traits and physical-function decline remains incompletely characterized. **Methods:** We applied genomic structural equation modeling to European-ancestry GWAS summary statistics for five constituent phenotypes: depressive symptoms, depression diagnosis, grip strength, appendicular lean mass, and walking pace. A Depression–Physical Function shared genetic factor was constructed as a cross-trait genetic covariance dimension and evaluated using LDSC-based validation and leave-one-trait-out sensitivity analyses. We then performed factor GWAS, FUMA locus annotation, Bayesian fine-mapping, MAGMA gene-based analysis, transcriptome-wide association analysis, pathway enrichment, CELLECT/MAGMA cell-type specificity analysis, partitioned heritability analysis, and gsMap spatial transcriptomic mapping. **Results:** The shared factor showed good model fit and retained 755,397 quality-controlled variants for downstream analysis. The factor was positively genetically correlated with depression-related traits and negatively correlated with physical-function-related traits. FUMA identified 245 genome-wide significant SNPs, 44 lead SNPs, and 38 genomic risk loci, with 127 positional mapped genes. Fine-mapping prioritized one high-confidence locus. MAGMA identified 19 Bonferroni-significant genes and 326 FDR-significant genes, while TWAS identified 322 FDR-significant expression-associated genes. Integrating FUMA positional mapping, MAGMA gene-level association and TWAS expression-level association prioritized eight convergent genes: TMEM106B, CENPW, DRD2, LRFN5, NCAPG, DCAF16, SGIP1, and FAM120A. Functional enrichment highlighted postsynaptic structure, neuron spine, synaptic plasticity, and synapse organization. CELLECT/MAGMA prioritized brain non-myeloid neurons and glial populations, with additional endocrine-metabolic and immune-hematopoietic signals. Spatial transcriptomic mapping localized top signals to brain and spinal cord regions in the embryonic neuro-muscle reference. Partitioned heritability analysis showed enrichment in conserved, intronic, promoter, and chromatin-related genomic annotations. **Conclusions:** These findings support a shared polygenic covariance dimension linking depression-related liability with reduced physical-function-related genetic propensity. Downstream analyses prioritized candidate loci, genes, and biological contexts, with enrichment patterns consistent with neuronal, synaptic, and regulatory genomic processes.

## 1. Introduction

Depression-related traits and reduced physical function are major determinants of health outcomes in middle-aged and older adults [1,2]. Depression is characterized by persistent low mood, loss of interest, impaired sleep and appetite, cognitive symptoms, and reduced social functioning, and is associated with disability [3], reduced quality of life, and increased mortality risk [4]. In parallel, reduced grip strength, lower appendicular lean mass, and slower walking pace are important indicators of declining physical capacity, mobility limitation, and reduced functional reserve [5]. Grip strength is widely used as a proxy for muscular fitness and frailty-related vulnerability, appendicular lean mass reflects the body-composition dimension of muscle-related function, and walking pace captures mobility and integrated physical performance [6]. Together, these phenotypes provide complementary measures of physical-function-related decline.

Clinical and epidemiological studies have consistently reported associations between depression-related symptoms and poorer physical function [7,8]. Lower grip strength and slower walking pace have been associated with increased risk of subsequent depressive symptoms, while depressive symptoms may also contribute to reduced activity, impaired sleep, lower appetite, social withdrawal, and progressive functional limitation [9,10]. These findings indicate that mood-related traits and physical-function-related traits are closely connected across psychological, behavioral, and somatic domains. However, observational associations alone cannot determine whether these phenotypes share a common genetic basis or whether their correlation mainly reflects downstream behavioral or disease-related processes.

Several biological processes may plausibly link depression-related liability with reduced physical function. Depression has been associated with neuroinflammation, oxidative stress, altered neuroendocrine regulation, impaired neurogenesis, and changes in synaptic plasticity [11,12]. Reduced muscle strength, lower lean mass, and mobility decline have also been linked to inflammation, mitochondrial dysfunction, impaired protein turnover, and reduced regenerative capacity [13,14]. These overlapping biological themes suggest that mood-related and physical-function-related phenotypes may share upstream mechanisms. A genetic framework can help clarify whether this cross-domain relationship is supported by shared polygenic architecture.

Previous genetic studies have examined depression and muscle-related traits using univariate GWAS, genetic correlation analysis, and Mendelian randomization [15,16]. These approaches have provided important evidence for heritability, genetic overlap, and potential directional relationships between selected phenotype pairs. Nevertheless, depression-related traits, grip strength, appendicular lean mass, and walking pace represent correlated but distinct phenotypic dimensions. Pairwise analyses cannot fully capture the higher-order genetic covariance structure shared across these traits. A multivariate genetic approach is therefore needed to model their common genetic component directly.

Genomic structural equation modeling provides a suitable framework for this purpose. By using GWAS summary statistics and linkage disequilibrium score regression-derived genetic covariance matrices, Genomic SEM can model latent genetic factors across multiple genetically correlated phenotypes and perform factor GWAS to identify variants associated with the shared component [17]. This approach has been applied to complex psychiatric and multidimensional traits to reveal genetic architecture that may not be fully captured by single-trait analyses.

In the present study, we integrated GWAS summary statistics for two depression-related phenotypes and three physical-function-related phenotypes to construct a Depression–Physical Function shared genetic factor. The depression-related domain included depressive symptoms and depression diagnosis, while the physical-function-related domain included grip strength, appendicular lean mass, and walking pace. We defined the factor as a summary-statistics-based genetic covariance dimension rather than a directly observed clinical syndrome or causal pathway. We then performed factor GWAS and multilayer post-GWAS analyses to characterize the shared polygenic covariance structure and prioritize candidate variants, genes, pathways, cell types, and functional genomic contexts for future validation.

## 2. Methods

### 2.1. GWAS Data Acquisition for Genomic SEM Analysis

**Figure 1:**
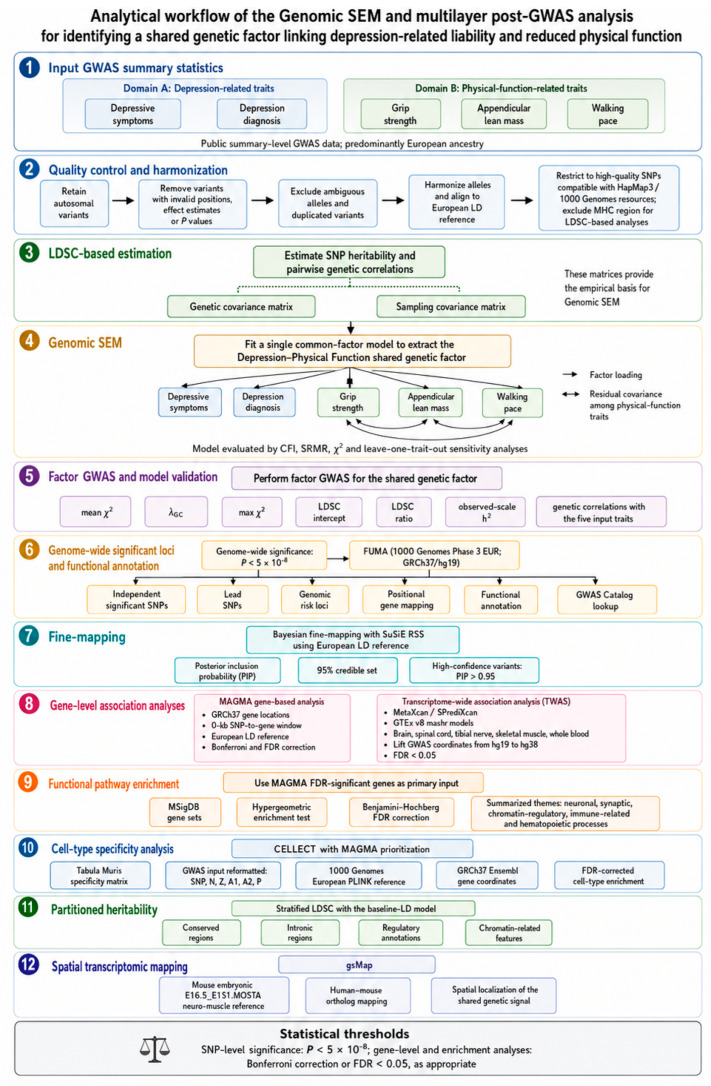
Analytical workflow of the Genomic SEM and multilayer post-GWAS analysis.

The overall analytical workflow of this study is shown in Figure 1. This study used publicly available genome-wide association study summary statistics to investigate the shared genetic architecture linking depression-related liability and reduced physical function. Five constituent phenotypes were selected to represent two major domains: depression-related traits and physical-function-related traits. The depression-related domain included depressive symptoms and depression diagnosis. The physical-function-related domain included grip strength, appendicular lean mass, and walking pace.

All analyses were conducted using summary-level GWAS data, and no individual-level genotype or phenotype data were used. The selected GWAS datasets were restricted to European-ancestry or predominantly European-ancestry participants to reduce confounding from population stratification. For each input GWAS, the public data source, phenotype definition, GWAS identifier, sample size, ancestry, phenotype type, and observed-scale SNP heritability used in this study are summarized in Supplementary Table S1.

The five traits were treated as genetically correlated proxy phenotypes rather than as a directly observed clinical comorbidity cohort. The purpose of the Genomic SEM analysis was to extract a latent genetic covariance factor linking depression-related liability with reduced physical-function-related genetic propensity. Because the input GWAS differed in phenotype definition, measurement scale, and cohort composition, these features were documented for each dataset before multivariable genetic modeling.

The workflow included GWAS summary statistic harmonization, LDSC-based genetic covariance estimation, Genomic SEM factor construction, factor GWAS, and multilayer post-GWAS analyses including FUMA, fine-mapping, MAGMA, TWAS, pathway enrichment, cell-type specificity analysis, partitioned heritability analysis, and spatial transcriptomic mapping.

### 2.2. Quality Control and Harmonization of GWAS Summary Statistics

Before Genomic SEM analysis, GWAS summary statistics were harmonized and filtered to ensure consistency across input traits. Autosomal variants were retained for downstream analyses. Variants with invalid chromosome or base-pair positions, invalid or missing effect estimates, invalid *p*-values, ambiguous allele coding, or duplicated variant identifiers were excluded. Alleles were harmonized across GWAS datasets, and variants were aligned to a European ancestry linkage disequilibrium reference panel [18].

For LDSC and Genomic SEM preparation, summary statistics were formatted to include variant identifier, chromosome, base-pair position, effect allele, non-effect allele, association statistic, and *p*-value. Variants were further restricted to high-quality SNPs compatible with the HapMap3 and 1000 Genomes Project European reference resources where required by LDSC or downstream tools [19]. The major histocompatibility complex region was excluded in LDSC-based analyses when required by the reference pipeline.

After harmonization, each input GWAS was converted into LDSC-compatible summary statistics. These harmonized files were used to estimate SNP heritability, pairwise genetic covariance, and sampling covariance among the five constituent traits.

### 2.3. Estimation of SNP Heritability and Pairwise Genetic Correlations

Linkage disequilibrium score regression was used to estimate SNP-based heritability and pairwise genetic correlations among the five constituent traits [20]. Multivariable LDSC was then used to generate the empirical genetic covariance matrix and the corresponding sampling covariance matrix required for Genomic SEM.

The diagonal elements of the genetic covariance matrix were interpreted as observed-scale SNP heritability estimates for each input trait, whereas the off-diagonal elements represented genetic covariance between trait pairs. The genetic covariance matrix was further standardized to obtain the pairwise genetic correlation matrix. These LDSC-derived matrices provided the empirical basis for subsequent Genomic SEM model fitting. Potential sample overlap across input GWAS was evaluated using the LDSC-derived sampling covariance matrix and cross-trait intercepts. Several input GWAS were derived wholly or partly from UK Biobank or related European-ancestry cohorts, and participant overlap could therefore not be excluded a priori. In Genomic SEM, the LDSC sampling covariance matrix was used to account for sampling error and potential sample overlap among summary statistics. Cross-trait intercepts and sampling-covariance diagnostics are reported in Supplementary Table S2 and were considered when interpreting the genetic covariance structure.

### 2.4. Construction of the Depression–Physical Function Shared Genetic Factor

Genomic structural equation modeling was used to model the genetic covariance structure across the five constituent traits. A single common-factor model was specified to extract a latent factor representing covariance shared by depression-related and physical-function-related traits. The factor was named the Depression–Physical Function shared genetic factor and is referred to hereafter as the shared genetic factor. It was interpreted as a bipolar genetic covariance dimension, not as a directly observed comorbid phenotype or causal pathway.

In the model, depressive symptoms, depression diagnosis, grip strength, appendicular lean mass, and walking pace were specified as indicators of the shared genetic factor. Residual covariance terms were allowed among grip strength, appendicular lean mass, and walking pace to account for additional genetic covariance within the physical-function-related domain. The latent factor variance was fixed to one for model identification, and the residual variance of depressive symptoms was fixed to zero as a boundary constraint because freely estimating this parameter produced a negative residual variance. A boundary sensitivity analysis was conducted by comparing the final constrained model with an unconstrained specification in which the depressive symptoms residual variance was freely estimated.

Model fit was evaluated using standard Genomic SEM fit indices, including comparative fit index, standardized root mean square residual, model chi-square, degrees of freedom, and Akaike information criterion. Factor loadings, residual covariance estimates, and residual variance estimates were reported with standard errors. Because the analysis was based on GWAS summary statistics and did not generate individual-level factor scores, factor determinacy was not estimated. To assess whether the latent factor structure was disproportionately driven by any single input phenotype, leave-one-trait-out sensitivity analyses were conducted by sequentially removing one constituent phenotype and refitting the model using the remaining traits.

### 2.5. Model Validation and Stability Assessment of the Factor GWAS

To evaluate the robustness and SNP-level heterogeneity of the factor GWAS, we performed LDSC-based validation, summary-level diagnostic assessment, and Genomic SEM Q_SNP analysis. The final factor GWAS summary statistics were assessed using multiple metrics, including the number of retained variants, mean chi-square statistic, genomic inflation factor, maximum chi-square statistic, LDSC intercept, LDSC ratio, and observed-scale SNP heritability. Q_SNP analysis was implemented using userGWAS with the final factor model, fixed measurement parameters, smooth checking enabled, and Q_SNP estimation requested. This analysis was applied to genome-wide significant SNPs and independent genome-wide significant lead SNPs to evaluate whether associated variants showed residual trait-specific heterogeneity beyond their association with the shared factor.

To further assess whether the lead factor-GWAS associations were disproportionately driven by any single constituent phenotype, we performed a lead-SNP-level leave-one-trait-out factor-GWAS sensitivity analysis. The 43 tested lead SNPs available for this analysis were re-evaluated after sequentially removing depressive symptoms, depression diagnosis, grip strength, appendicular lean mass, or walking pace from the factor model. For each leave-one-trait-out specification, SNP effect estimates, effect directions, and association *p*-values were compared with those from the primary all-trait model.

The LDSC ratio was calculated as:

$$\mathrm{LDSC}\;\mathrm{ratio}=\frac{\mathrm{LDSC}\;\mathrm{intercept}-1}{\mathrm{mean}\;\chi^{2}-1}$$

This metric was used to evaluate the extent to which inflation in GWAS test statistics could be attributed to residual confounding rather than polygenic signal. Observed-scale SNP heritability of the factor GWAS was estimated by LDSC. Genetic correlations between the shared genetic factor and each of the five input traits were also estimated to examine whether the factor captured the expected direction of association with depression-related and physical-function-related phenotypes.

### 2.6. Identification and Functional Annotation of Genome-Wide Significant Loci

Genome-wide significant SNPs were defined using the conventional threshold of *p* < 5 × 10^−8^. Functional mapping and annotation of genome-wide association studies was used to define independent significant SNPs, lead SNPs, and genomic risk loci from the factor GWAS summary statistics [21].

FUMA analysis was performed using the 1000 Genomes Project Phase 3 European reference panel and the GRCh37/hg19 genome build. Independent significant SNPs and lead SNPs were defined according to linkage disequilibrium thresholds implemented in FUMA. Genomic risk loci were defined by merging physically close lead SNP regions according to the default or prespecified FUMA locus-merging distance. Positional gene mapping was performed to assign candidate genes to genome-wide significant loci.

FUMA outputs included independent significant SNPs, lead SNPs, genomic risk loci, candidate SNP annotation, positional mapped genes, functional annotation categories, and GWAS Catalog [22] lookup results. These outputs were used to summarize the variant- and locus-level architecture of the shared genetic factor.

### 2.7. Fine-Mapping of Associated Loci

Bayesian fine-mapping was performed for genome-wide significant loci to prioritize candidate variants within associated regions. Genomic risk loci defined by FUMA were used as the locus-level input for fine-mapping.

For each locus, a window centered on the lead SNP was extracted from the factor GWAS summary statistics. Linkage disequilibrium matrices were generated using a European ancestry reference panel. Fine-mapping was performed using summary-statistics-based Bayesian fine-mapping with SuSiE RSS [23]. For each locus, posterior inclusion probabilities were estimated for variants within the locus window, and 95% credible sets were generated where supported by the data.

A variant was considered high-confidence if it achieved posterior inclusion probability greater than 0.95. Fine-mapping results were summarized at the locus level, including lead SNP, locus coordinates, number of overlapping variants, credible set size, top posterior probability SNP, and high-confidence status. Fine-mapping was interpreted as a prioritization analysis rather than definitive proof of causality.

### 2.8. Gene-Level and Transcriptome-Wide Association Methods

Gene-level association analyses were conducted using two complementary approaches: MAGMA gene-based analysis and transcriptome-wide association analysis.

#### 2.8.1. Magma Gene-Based Analysis

MAGMA was used to perform gene-based association testing based on the factor GWAS summary statistics [24]. SNP-level *p*-values were mapped to genes using a GRCh37 gene location file. A 0 kb gene window was used for SNP-to-gene annotation. Gene-level association tests were performed using a European ancestry linkage disequilibrium reference panel. Because the factor GWAS summary statistics used a fixed sample size field, MAGMA was run with a conservative effective sample size of 322,580, corresponding to the smallest sample size among the five input GWAS after harmonization.

MAGMA gene-level results were corrected for multiple testing using both Bonferroni correction and Benjamini–Hochberg false discovery rate correction. Bonferroni-significant genes were used to identify the most conservative gene-level signals, whereas FDR-significant genes were used as the primary input for downstream pathway enrichment analysis.

#### 2.8.2. Transcriptome-Wide Association Analysis

Transcriptome-wide association analysis was performed to identify genes whose genetically regulated expression was associated with the shared genetic factor. The analysis used MetaXcan/SPrediXcan with GTEx v8 mashr expression prediction models [25,26]. Tissue models were selected to cover brain regions, spinal cord, tibial nerve, skeletal muscle, and whole blood.

Because the expression prediction models used genome build hg38 variant identifiers, factor GWAS coordinates were lifted from hg19 to hg38 before TWAS. Variant identifiers were reformatted to match the GTEx v8 model format, and allele orientation was checked before analysis. Tissue-specific TWAS results were generated for each selected tissue model, and FDR correction was applied across all gene-tissue tests. For gene-level reporting, the strongest tissue-specific TWAS signal for each gene was selected according to the smallest TWAS *p*-value. FOCUS was used as an additional transcriptome-wide prioritization layer. No formal GWAS-eQTL colocalization, SMR, or HEIDI analysis was performed.

### 2.9. Functional Pathway Enrichment Analysis

Functional enrichment analysis was performed using MAGMA FDR-significant genes as the primary input gene set. The background set consisted of all genes tested in the MAGMA gene-based analysis.

Gene-set over-representation analysis was performed using curated human gene sets from the Molecular Signatures Database [27,28]. Gene identifiers were harmonized to Entrez IDs, and only gene sets with sufficient overlap with the MAGMA-tested background were retained. Hypergeometric enrichment testing was used to evaluate whether MAGMA FDR-significant genes were over-represented in each gene set. Multiple testing correction was performed using the Benjamini–Hochberg method, and enriched pathways were considered significant at FDR < 0.05. Pathway enrichment results were summarized by biological theme, with emphasis on neuronal, synaptic, chromatin-regulatory, immune-related, and hematopoietic processes.

### 2.10. Cell-Type Specificity Analysis Methods

Cell-type specificity analysis was performed using CELLECT with MAGMA prioritization [29]. The purpose of this analysis was to evaluate whether genes with cell-type-specific expression patterns showed disproportionate association with the shared genetic factor.

A Tabula Muris cell-type specificity matrix was used as the single-cell reference resource [30]. The factor GWAS summary statistics were reformatted for CELLECT/MAGMA, including SNP identifier, sample size, Z statistic, effect allele, non-effect allele, and *p*-value. The Z statistic was calculated from the GWAS effect estimate and standard error. A 1000 Genomes European PLINK reference panel and a GRCh37 Ensembl gene coordinate file were used for MAGMA annotation within the CELLECT workflow.

CELLECT prioritization was performed across Tabula Muris cell-type annotations. MAGMA gene-level statistics were corrected for internal gene properties, and corrected gene-level statistics were regressed against cell-type specificity annotations. Cell-type enrichment significance was evaluated using FDR correction across all tested cell types. Cell types with FDR < 0.05 were considered significant.

The final CELLECT workflow used a prepared GWAS input with 755,397 variants before MAGMA matching; the input columns were SNP, N, Z, A1, A2, and PVAL. The Tabula Muris specificity matrix, GRCh37 Ensembl v91 gene coordinates, and a 1000 Genomes European PLINK reference were used in the successful workflow.

### 2.11. Partitioned Heritability Analysis

Stratified LDSC was used to partition the SNP heritability of the shared genetic factor across functional genomic annotations [31]. The baseline-LD model was used to evaluate enrichment across broad functional categories, including conserved regions, coding regions, regulatory annotations, and chromatin-related features [32].

For each annotation, stratified LDSC estimated the coefficient, enrichment, standard error, *p*-value, and FDR-adjusted *p*-value. Annotations with FDR < 0.05 were considered significantly enriched. This analysis was used to evaluate whether heritability of the shared genetic factor was preferentially localized to specific functional genomic compartments.

### 2.12. Spatial Transcriptomic Mapping Methods

Spatial transcriptomic mapping was performed to localize the genetic signal of the shared genetic factor within tissue-level spatial gene-expression contexts. The analysis used gsMap to integrate the factor GWAS summary statistics with mouse embryonic spatial transcriptomic data [33].

A mouse embryonic E16.5_E1S1.MOSTA neuro-muscle spatial reference was used for spatial mapping. Human–mouse ortholog mapping was used to connect human GWAS-associated genes with mouse spatial gene-expression profiles. The workflow generated spatial mapping statistics, gene diagnostic outputs, and spatial visualization files.

### 2.13. Statistical Significance and Reporting

For SNP-level association testing, genome-wide significance was defined as *p* < 5 × 10^−8^. For gene-level and enrichment analyses, multiple testing was controlled using Bonferroni correction or Benjamini–Hochberg FDR correction as appropriate. MAGMA gene-level results were reported using both Bonferroni-significant and FDR-significant thresholds. Pathway, TWAS, cell-type, and partitioned heritability analyses used FDR < 0.05 as the primary significance threshold, and post-GWAS enrichment and mapping results were interpreted as exploratory prioritization evidence.

All analyses were based on summary-level data from previously published GWAS or publicly available reference resources. Therefore, no new individual-level human participant data were collected, and no additional ethical approval was required for the present secondary analysis.

## 3. Results

### 3.1. Structural Equation Model Fitting

LDSC analysis showed that all five constituent GWAS contributed measurable SNP-based heritability to the multivariate modeling framework. Observed-scale SNP heritability estimates were 0.042 for depressive symptoms, 0.075 for depression diagnosis, 0.120 for grip strength, 0.366 for appendicular lean mass, and 0.073 for walking pace (Table 1).

**Figure 2:**
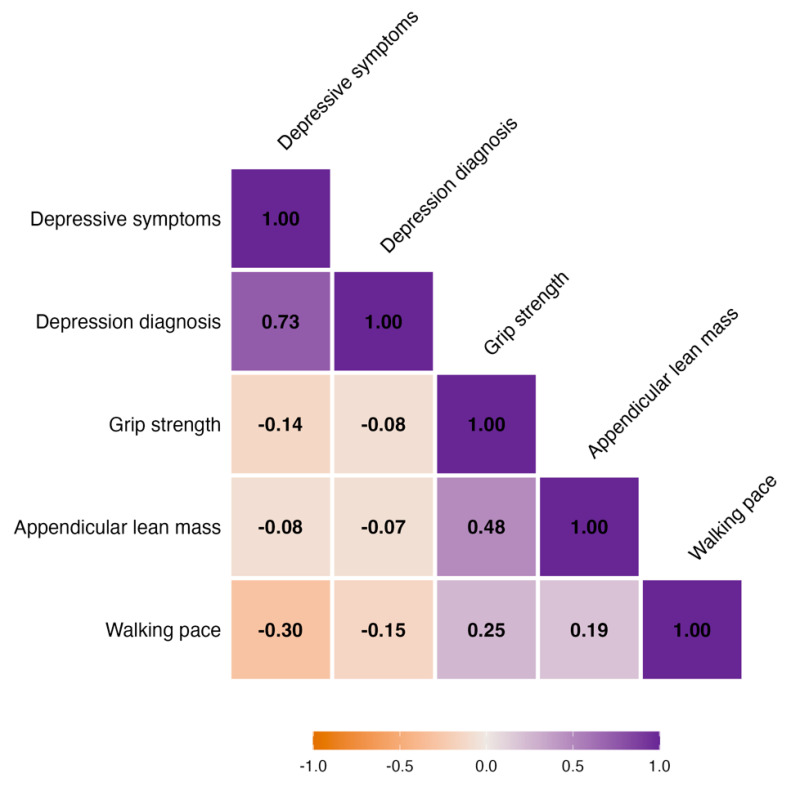
Pairwise genetic correlation structure among the five constituent phenotypes.

The pairwise genetic correlation matrix showed a coherent correlation structure among the five constituent phenotypes (Figure 2). The two depression-related traits were positively correlated at the genetic level (rg = 0.734). Depression-related traits were generally negatively correlated with physical-function-related traits. The strongest inverse genetic correlation was observed between depressive symptoms and walking pace (rg = −0.303), followed by depressive symptoms and grip strength (rg = −0.144), depression diagnosis and walking pace (rg = −0.154), and weaker inverse correlations involving appendicular lean mass. In contrast, the three physical-function-related traits showed positive genetic correlations, especially grip strength with appendicular lean mass (rg = 0.480), followed by grip strength with walking pace (rg = 0.250) and appendicular lean mass with walking pace (rg = 0.188). These results supported the presence of detectable but modest shared genetic covariance between depression-related liability and reduced physical-function-related traits.

A single shared genetic factor was then fitted to the LDSC-derived genetic covariance matrix. The final constrained model showed good overall fit, with CFI = 0.9958, SRMR = 0.0175, χ^2^ = 14.15, df = 3, and AIC = 38.15 (Table 2). In the boundary sensitivity analysis, freely estimating the depressive symptoms residual variance produced a negative estimate (estimate = −0.0132, SE = 0.00636, *p* = 0.0382), supporting the use of the zero-boundary constraint in the final model. The standardized factor loadings were positive for depressive symptoms and depression diagnosis and negative for grip strength, appendicular lean mass, and walking pace, consistent with the direction of the predefined shared genetic dimension. Leave-one-trait-out analyses showed that the shared factor remained identifiable after sequential removal of each constituent phenotype, with the expected loading direction retained across interpretable submodels. Full factor loadings, residual covariance estimates, and residual variance estimates are provided in Supplementary Table S3. Model comparison and leave-one-trait-out results are summarized in Table 2 and Supplementary Table S4.

### 3.2. Stability Assessment of the Shared Genetic Factor GWAS

The factor GWAS for the Depression–Physical Function shared genetic factor retained 755,397 quality-controlled variants for downstream analyses. LDSC-based validation indicated a mean χ^2^ of 1.289 and λGC of 1.198, consistent with moderate enrichment of association statistics. The maximum χ^2^ was 70.09. The LDSC intercept was 0.934, and the ratio estimate was negative, suggesting that the observed inflation was unlikely to be explained primarily by residual confounding, although these LDSC diagnostics should be interpreted cautiously because intercepts below one and negative ratios may reflect estimation variability. The observed-scale SNP heritability of the shared factor was 0.0552, corresponding to an h^2^ Z score of approximately 17.3. These metrics supported sufficient polygenic signal for downstream genetic and functional analyses.

The shared factor showed the expected direction of genetic association with the five input traits. It was positively correlated with depression-related phenotypes and negatively correlated with physical-function-related phenotypes, supporting its interpretation as a genetic dimension linking depression-related liability with reduced physical function.

**Figure 3:**
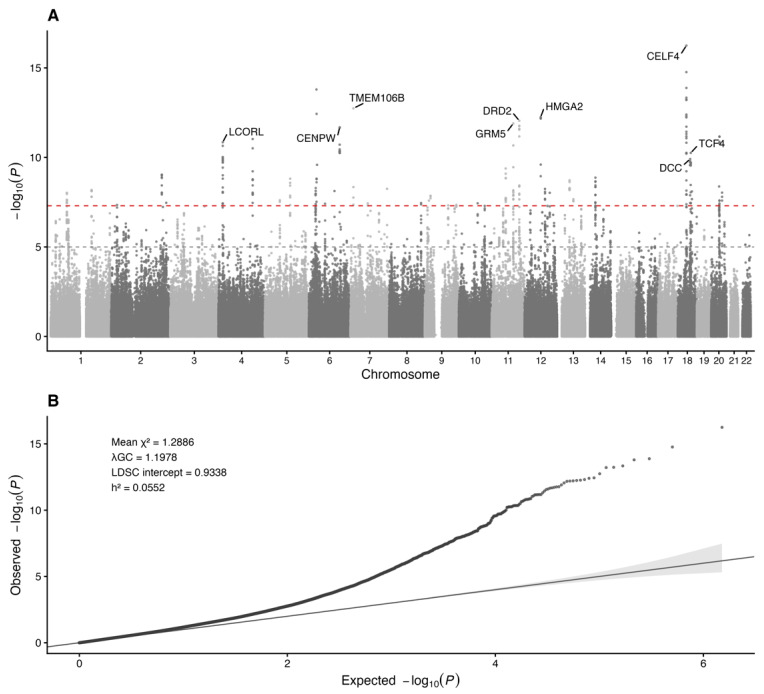
Genome-wide association results for the Depression–Physical Function shared genetic factor. (**A**) Manhattan plot; (**B**) quantile–quantile plot. In the Manhattan plot, the red dashed horizontal line indicates the genome-wide significance threshold; in the quantile–quantile plot, the grey band indicates the expected confidence interval under the null distribution.

A Manhattan plot and quantile–quantile plot were generated to visualize the genome-wide distribution of association statistics (Figure 3). The Manhattan plot showed multiple genome-wide significant regions across the genome, while the QQ plot was consistent with polygenic enrichment rather than isolated inflation.

### 3.3. Genome-Wide Significant Loci and Functional Annotation

The factor GWAS identified 245 SNPs reaching genome-wide significance at *p* < 5 × 10^−8^. FUMA delineated these signals into 71 independent significant SNPs, 44 lead SNPs, and 38 genomic risk loci. Positional mapping assigned 127 genes to these genomic risk loci. The leading mapped genes ranked by minimum GWAS *p*-value included CELF4, TMEM106B, HMGA2, DRD2, GRM5, UQCC1, MAML3, LCORL, CENPW, TCF4, and DCC.

To further evaluate whether these factor-GWAS signals reflected homogeneous effects through the shared factor, we performed Genomic SEM Q_SNP analysis for genome-wide significant SNPs and independent lead SNPs. Among 679 genome-wide significant SNPs available for Q_SNP testing, 561 showed Q_SNP FDR < 0.05. Among 43 tested lead SNPs, 33 showed Q_SNP FDR < 0.05. These results indicate that many genome-wide significant factor-GWAS signals showed evidence of residual SNP-level heterogeneity, suggesting that associated loci may include trait-specific components in addition to association with the shared genetic factor.

We next evaluated the stability of the 43 lead SNP associations across lead-SNP-level leave-one-trait-out factor-GWAS specifications. All leave-one-trait-out models were successfully fitted at the lead-SNP level. The SNP effect estimates remained highly correlated with the main all-trait model across the five leave-one-trait-out specifications, with beta correlations ranging from 0.8394 to 0.9977. All 43 lead SNPs retained the same direction of effect across all leave-one-trait-out models, and 39 of 43 lead SNPs remained nominally significant in every leave-one-trait-out specification. No lead SNP showed a direction flip after removal of any single constituent phenotype. These results suggest that the lead factor-GWAS associations were not driven solely by one individual input phenotype.

The genomic risk loci were distributed across multiple chromosomes, including prominent association regions on chromosomes 4, 6, 7, 11, 12, 18, and 20. Functional annotation of candidate SNPs showed that many mapped variants were located in intronic and regulatory-proximal categories, including 3′ untranslated regions, 5′ untranslated regions, upstream regions, and intronic regions.

### 3.4. Fine-Mapping of Associated Loci

Bayesian fine-mapping was performed for all 38 FUMA-defined genomic risk loci. All 38 loci were successfully processed. One locus reached the predefined high-confidence criterion of posterior inclusion probability greater than 0.95 within the 95% credible set. This locus was located on chromosome 11, with lead SNP rs627387 at position 88,694,072. The top posterior probability SNP was rs1000061, with posterior inclusion probability = 1.00, and the credible set contained 10 variants. The remaining loci did not contain variants meeting the high-confidence posterior probability threshold.

### 3.5. Gene-Level Association Analysis

#### 3.5.1. Magma Gene-Level Association

**Figure 4:**
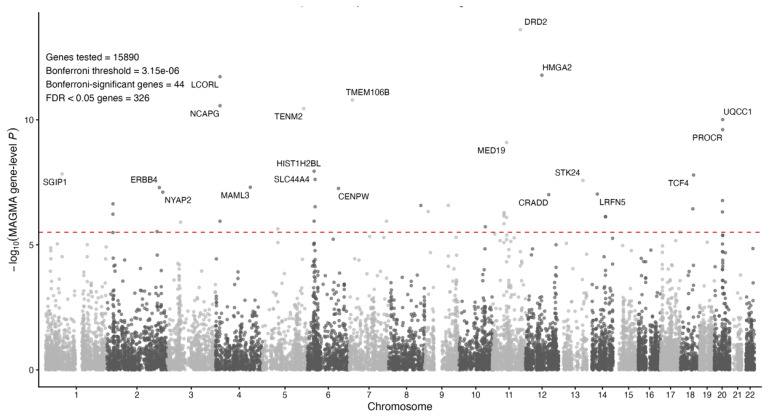
MAGMA gene-level association results for the Depression–Physical Function shared genetic factor. The red dashed horizontal line indicates the Bonferroni-corrected gene-level significance threshold, and labelled points indicate top-ranked genes.

MAGMA gene-based association analysis tested 15,890 genes. Using Bonferroni correction, 19 genes reached gene-level significance. Using FDR < 0.05, 326 genes were identified as FDR-significant and were used as the primary gene set for downstream pathway enrichment analysis. The top-ranked MAGMA genes included DRD2, HMGA2, LCORL, TMEM106B, NCAPG, TENM2, UQCC1, PROCR, MED19, HIST1H2BL, SGIP1, TCF4, SLC44A4, STK24, MAML3, ERBB4, CENPW, and NYAP2. The MAGMA gene-level association results are shown in Figure 4.

#### 3.5.2. TWAS Expression-Level Association

**Figure 5:**
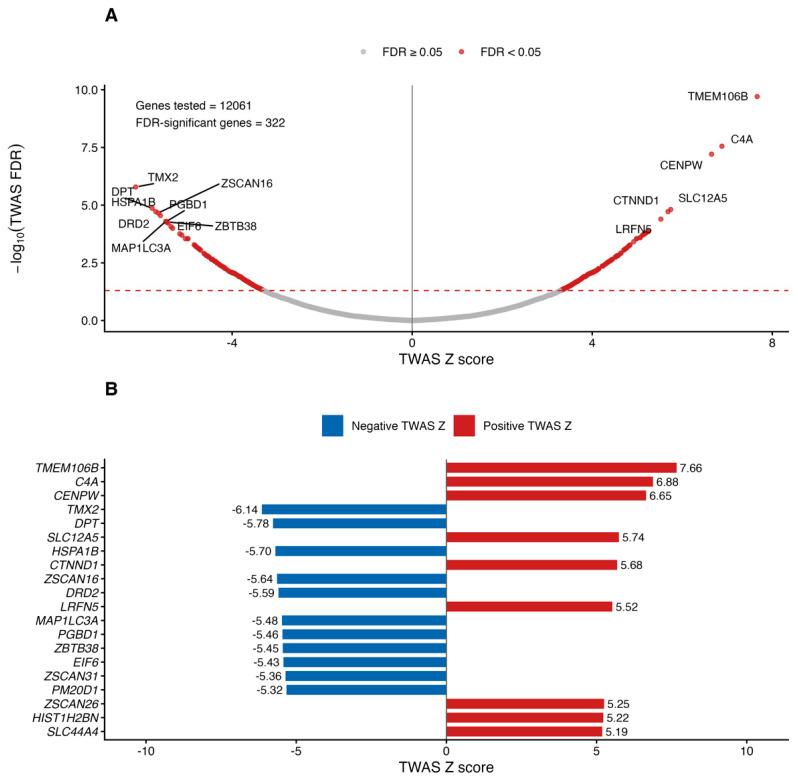
Transcriptome-wide association analysis of the Depression–Physical Function shared genetic factor. (**A**) Volcano plot of TWAS results. (**B**) Top TWAS-associated genes ranked by TWAS Z score. In (**A**), red and blue points indicate positive and negative TWAS associations, respectively, and the dashed horizontal line indicates the FDR significance threshold. In (**B**), bars show the top TWAS-associated genes ranked by TWAS Z score, with red and blue bars indicating positive and negative TWAS Z scores, respectively.

TWAS was performed across 13 GTEx v8 mashr tissue models covering brain regions, spinal cord, tibial nerve, skeletal muscle, and whole blood. Across 46,487 gene-tissue tests, 912 gene-tissue associations reached FDR < 0.05, corresponding to 322 unique TWAS-supported genes. The strongest TWAS signals included TMEM106B, C4A, and CENPW, with additional significant expression-level associations observed for genes overlapping MAGMA or FUMA-supported regions, including NCAPG, DRD2, TMEM106B, and CENPW. The TWAS results are summarized in Figure 5. Genes supported by convergent locus-, gene-, and expression-level evidence are listed in Table 3.

### 3.6. Pathway Enrichment Analysis

**Figure 6:**
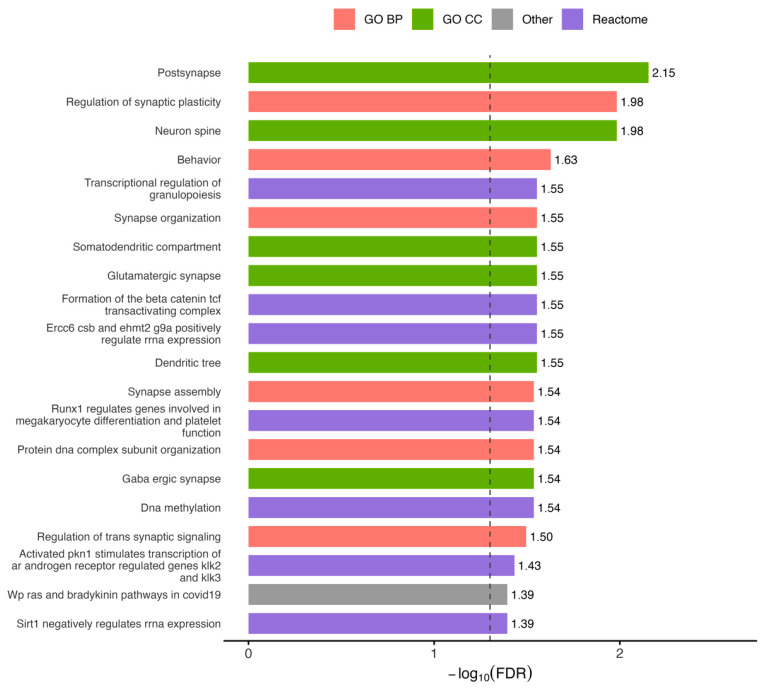
Functional pathway enrichment analysis of MAGMA FDR-significant genes. Bar colors indicate pathway categories, and the vertical dashed line indicates the FDR significance threshold.

To characterize the biological processes represented by genes associated with the Depression–Physical Function shared genetic factor, we performed functional pathway enrichment analysis based on MAGMA FDR-significant genes. After excluding the strongest outlier term to improve visualization, the top enriched pathways were dominated by neuronal and synaptic annotations (Figure 6). These included postsynapse, neuron spine, regulation of synaptic plasticity, synapse organization, glutamatergic synapse, dendritic tree, and somatodendritic compartment. Enrichment was also observed for behavior and regulation of trans-synaptic signaling, indicating that neural-communication-related annotations were prioritized in this gene-set analysis.

In addition to these synaptic terms, several pathways related to transcriptional and epigenetic regulation were present among the top enriched results, including DNA methylation, protein–DNA complex subunit organization, and Reactome terms related to rRNA expression regulation and β-catenin/TCF transcriptional complex formation. These results suggest that the gene-level signals underlying the shared genetic factor are not restricted to individual loci, but prioritize broader biological programs related to neuronal structure, synaptic organization, synaptic plasticity, and transcriptional regulation.

### 3.7. Cell-Type Prioritization Results

CELLECT/MAGMA cell-type prioritization was performed using Tabula Muris cell-type specificity annotations. A total of 115 cell-type annotations were tested, and 9 reached FDR < 0.05. The strongest cell-type prioritization signal was observed for brain non-myeloid neurons (β = 0.381, *p* = 3.31 × 10^−17^, FDR = 3.81 × 10^−15^). Additional significant brain-related cell types included brain non-myeloid oligodendrocyte precursor cells (FDR = 1.07 × 10^−3^), brain non-myeloid oligodendrocytes (FDR = 1.93 × 10^−2^), and brain non-myeloid astrocytes (FDR = 2.24 × 10^−2^).

**Figure 7:**
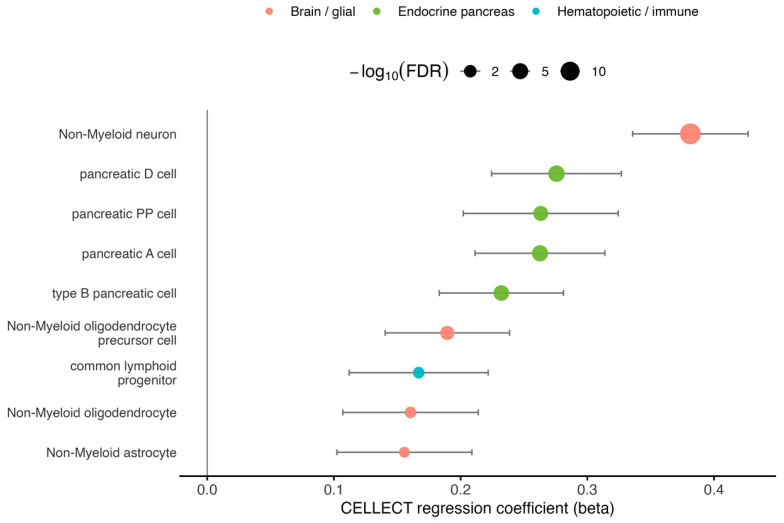
FDR-significant cell types prioritized by CELLECT/MAGMA. Only the 9 cell types surviving FDR correction are shown. Points indicate CELLECT regression coefficients, horizontal lines indicate standard errors, point size represents −log10(FDR), and colors indicate broad cell-type categories.

Beyond neuronal and glial cell types, significant enrichment was also observed for pancreatic endocrine cell types, including pancreatic D cells, pancreatic A cells, type B pancreatic cells, and pancreatic PP cells. Marrow common lymphoid progenitors also reached FDR significance. In contrast, skeletal muscle satellite cells were not significantly enriched (*p* = 0.293, FDR = 1.000). Together, these results suggested that the shared genetic factor was more strongly linked to neuronal, glial, endocrine-metabolic, and hematopoietic/immune-related cellular contexts than to a skeletal-muscle-satellite-cell-specific pattern. The FDR-significant cell types are visualized in Figure 7.

### 3.8. Spatial Transcriptomic Mapping

Spatial transcriptomic mapping was performed using the E16.5_E1S1.MOSTA neuro-muscle spatial reference. The gsMap workflow generated spatial-LDSC statistics and annotated spatial mapping outputs for the Depression–Physical Function shared genetic factor. The top-ranked spatial spots showed strong association signals, with the leading spot annotated as Brain (spot 583_290; β = 2.51 × 10^−9^, SE = 3.34 × 10^−10^, Z = 7.53, *p* = 2.59 × 10^−14^). The next top-ranked spots were also predominantly annotated as Brain, including spots 571_288, 571_282, 675_228, 669_224, and 673_235.

**Figure 8:**
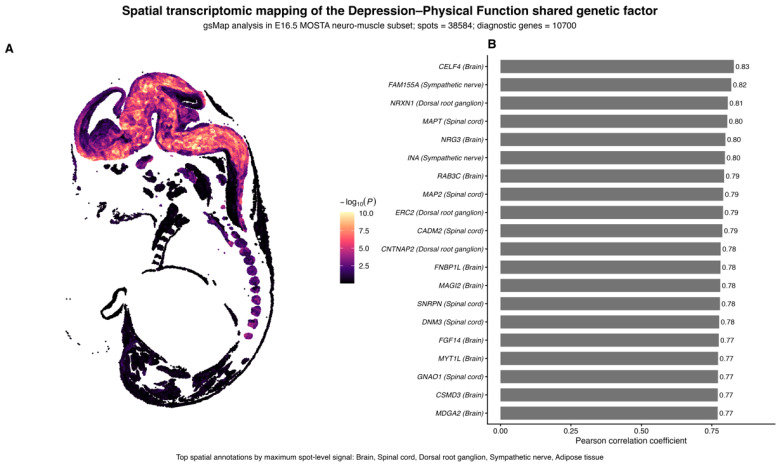
Spatial transcriptomic mapping of the shared genetic factor in the E16.5_E1S1.MOSTA neuro-muscle reference. (**A**) Spatial distribution of association signals across the embryonic neuro-muscle reference, with colors indicating −log10(P). (**B**) Top spatial annotations ranked by maximum spatial-signal strength.

Among the top 20 spatial spots, 17 were annotated as brain and 3 were annotated as spinal cord. Across the top 200 spatial mapping records, 171 spots were annotated as brain and 29 were annotated as spinal cord. The strongest spinal cord-associated spot was spot 598_312 (β = 3.01 × 10^−9^, SE = 4.14 × 10^−10^, Z = 7.27, *p* = 1.86 × 10^−13^). These results indicated that the strongest spatial prioritization signals mapped to brain and spinal cord regions of the embryonic neuro-muscle reference. The spatial mapping results are shown in Figure 8.

### 3.9. Partitioned Heritability Across Functional Genomic Annotations

**Figure 9:**
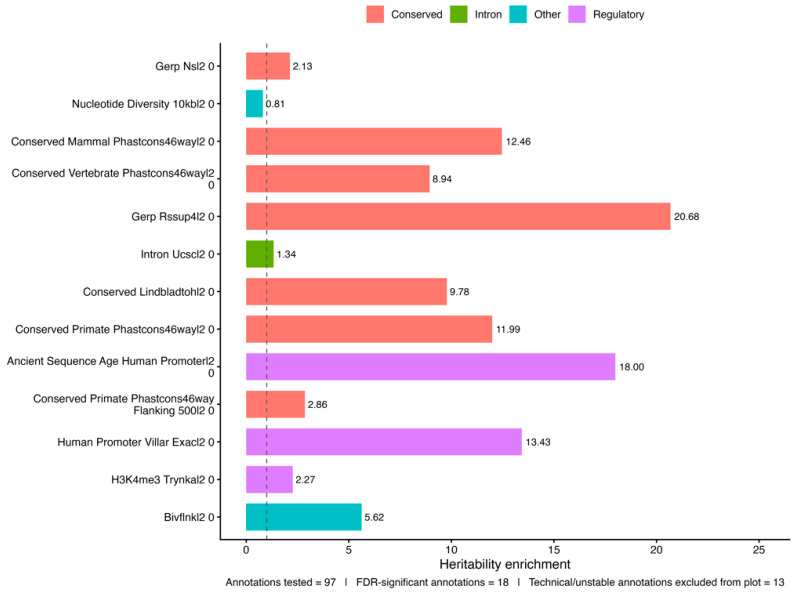
Partitioned heritability enrichment across functional genomic annotations.

Stratified LDSC was used to assess whether SNP heritability of the shared genetic factor was enriched across functional genomic annotations. A total of 97 annotations were tested, and 18 reached FDR < 0.05 (Figure 9). Significant enrichment was observed in several conserved sequence annotations, including Conserved Mammal phastCons 46-way, Conserved Vertebrate phastCons 46-way, GERP.RSsup4, Conserved Lindblad-Toh, and Conserved Primate phastCons 46-way. Regulatory and gene-structure-related annotations, including Intron UCSC, Ancient Sequence Age Human Promoter, Human Promoter Villar ExAC, H3K4me3 Trynka, and BivFlnk, also reached FDR significance. These results suggest that the SNP heritability of the shared genetic factor was preferentially localized to evolutionarily constrained, intronic, promoter and chromatin-related genomic regions.

Some significant annotations, such as MAF-adjusted, recombination-related, and background-selection-related features, were treated as genomic-architecture or technical covariates rather than directly interpretable biological pathways. Therefore, biological interpretation focused primarily on conserved sequence and regulatory annotations. Together, these findings indicate that the shared genetic architecture was not randomly distributed across the genome, but showed enrichment in functionally constrained and gene-regulatory genomic elements.

FDR-significant biologically interpretable annotations are shown after excluding technical or unstable annotations from visualization.

### 3.10. Integrated Multilayer Evidence

Overall, the downstream analyses provided a multilevel map of the genetic architecture underlying the Depression–Physical Function shared genetic factor. Variant- and locus-level analyses identified genome-wide significant regions and 127 positional mapped genes. Fine-mapping prioritized one high-confidence locus at variant resolution. Gene-level analyses highlighted both MAGMA-significant genes and TWAS expression-associated genes, including several genes overlapping neuropsychiatric and physical-function-related loci. Pathway enrichment further converged on neuronal and synaptic processes, particularly postsynaptic structure, neuron spine, synaptic plasticity, dendritic organization, and glutamatergic synapse. Cell-type analysis prioritized brain non-myeloid neurons and glial cell types, with additional endocrine-metabolic and hematopoietic/immune-related signals. Spatial transcriptomic mapping further localized top signals to brain and spinal cord regions within the embryonic neuro-muscle spatial reference. Partitioned heritability analysis supported enrichment in conserved, intronic, promoter and chromatin-related genomic annotations.

These findings support the interpretation that the shared genetic factor linking depression-related liability and reduced physical function is not driven by a single gene or single tissue system. Instead, it prioritizes a distributed set of neuronal, synaptic, glial, endocrine-metabolic, immune-related, and conserved regulatory genomic contexts for future investigation.

## 4. Discussion

This study used Genomic SEM to characterize a shared genetic covariance dimension linking depression-related liability with reduced physical-function-related genetic propensity. By integrating GWAS summary statistics for depressive symptoms, depression diagnosis, grip strength, appendicular lean mass, and walking pace, we constructed a Depression–Physical Function shared genetic factor and performed factor GWAS followed by multilayer post-GWAS analyses. The shared factor showed good model fit, and lead-SNP-level leave-one-trait-out sensitivity analyses indicated that the main lead factor-GWAS associations generally retained consistent effect directions after removal of individual constituent phenotypes. LDSC-based validation further indicated measurable SNP heritability and a polygenic signal suitable for downstream analysis. Its correlation pattern supported interpretation as a summary-statistics-based genetic covariance factor, although the pairwise genetic correlations were modest and should not be interpreted as evidence of a clinical syndrome, strong shared liability, or directional relationship.

The construction of this shared factor provides a summary-statistics-based framework for studying genetic covariance between psychiatric liability and physical-function-related phenotypes. The five input traits were selected as genetically correlated proxy phenotypes representing two domains: depression-related traits and physical-function-related traits. This design allowed cross-domain covariance to be modelled directly at the genetic level. The resulting shared genetic factor captures a latent polygenic covariance component that can be used for locus discovery and exploratory biological prioritization. Because the analysis was based on GWAS summary statistics, it does not stratify the shared factor by age, sex, or depression duration at the individual level.

At the variant and locus level, the factor GWAS identified multiple genome-wide significant regions. FUMA delineated these signals into independent significant SNPs, lead SNPs and genomic risk loci, and positional mapping assigned candidate genes to associated loci. The Q_SNP analysis further indicated that many genome-wide significant and lead SNPs showed residual SNP-level heterogeneity, suggesting that these loci should not be interpreted as acting exclusively through a homogeneous shared-factor pathway. Bayesian fine-mapping provided additional resolution for associated loci and prioritized one high-confidence locus with posterior inclusion probability greater than 0.95. Most loci remained unresolved at single-variant resolution, which is consistent with the complexity of polygenic traits and with the limited resolution of summary-statistics fine-mapping in regions with linkage disequilibrium [23].

Gene-level analyses provided a complementary layer of prioritization. MAGMA identified 19 Bonferroni-significant genes and 326 FDR-significant genes, while TWAS identified expression-associated genes across selected GTEx v8 tissue models. These two approaches capture different biological dimensions: MAGMA aggregates SNP-level association within gene boundaries, whereas TWAS links GWAS signal to genetically predicted gene expression. Integrating FUMA positional mapping, MAGMA gene-level association and TWAS expression-level association prioritized eight convergent genes, including TMEM106B, CENPW, DRD2, LRFN5, NCAPG, DCAF16, SGIP1, and FAM120A. These genes represent candidates supported by multiple analytical layers and provide a focused set of targets for future functional interpretation.

Several of these prioritized genes have plausible relevance to neuropsychiatric or neurobiological processes. DRD2 is a well-established dopamine receptor gene with broad relevance to reward processing, motivation, motor regulation, and psychiatric phenotypes [34,35]. TMEM106B has been implicated in lysosomal biology and neurodegenerative or brain-related traits [36]. LRFN5 is involved in neuronal connectivity and synaptic organization [37]. SGIP1 and DCAF16 also emerged through convergent gene-level evidence, while CENPW, NCAPG, and FAM120A may reflect broader cellular or regulatory mechanisms captured by the shared factor [38,39]. These gene-level findings should be interpreted as prioritization evidence rather than direct causal confirmation or proof of colocalized regulatory mechanisms, but they provide biologically interpretable entry points for subsequent validation.

Functional enrichment analysis of MAGMA FDR-significant genes revealed a prominent neuronal and synaptic pattern. After excluding the top outlier term from biological interpretation, the remaining FDR-significant pathways included postsynapse, neuron spine, regulation of synaptic plasticity, dendritic tree, somatodendritic compartment, synapse organization, glutamatergic synapse, and GABAergic synapse. These results suggest that the shared genetic factor is enriched for genes involved in neuronal structure, postsynaptic organization, and synaptic signaling [11]. This pathway-level pattern is consistent with central-nervous-system-related annotations being prominent within the shared genetic signal, but it should be interpreted as pathway prioritization rather than direct mechanistic evidence.

The enrichment of synaptic and neuronal pathways is biologically plausible. Depression-related phenotypes are strongly linked to neural circuits involved in affective regulation, reward processing, stress response, psychomotor activity, and motivation [40]. Physical-function-related traits such as grip strength and walking pace are influenced not only by peripheral muscle mass but also by motor planning, neuromuscular coordination, balance-related control, activity level, and central drive [41]. The pathway findings are therefore compatible with a brain−body framework for interpreting the shared genetic signal, while remaining hypothesis-generating rather than mechanistically conclusive.

Cell-type specificity analysis provided an additional prioritization layer for interpreting the shared genetic signal. CELLECT/MAGMA prioritization identified brain non-myeloid neurons as the strongest prioritized cell type, with additional significant enrichment in oligodendrocyte precursor cells, oligodendrocytes, and astrocytes. This pattern points to neuronal and glial cellular contexts as candidate contexts for follow-up. Oligodendrocyte-related signals may be relevant to myelination and neural conduction, while astrocyte-related signals may reflect metabolic support, synaptic homeostasis, and neuroimmune regulation [42,43]. Together with the pathway results, the cell-type findings support a hypothesis-generating cellular interpretation rather than direct cell-type-specific mechanism assignment.

Additional cell-type enrichment was observed in pancreatic endocrine cell types and marrow common lymphoid progenitors. These findings broaden the biological context of the shared factor beyond the central nervous system. Pancreatic endocrine signals may reflect metabolic regulation, energy balance, or systemic endocrine processes relevant to both depression-related traits and physical function [44]. The marrow common lymphoid progenitor signal suggests an immune-hematopoietic component, which is compatible with evidence linking inflammatory and immune pathways to mood symptoms and physical decline [45,46]. These non-neural signals were secondary to the dominant brain neuronal enrichment, but they indicate that the shared genetic architecture may involve systemic physiological regulation.

The absence of significant enrichment in skeletal muscle satellite cells is also informative. The physical-function-related traits included in the model capture muscle strength, lean mass, and walking pace, yet the cell-type enrichment signal did not concentrate in skeletal-muscle satellite cells within the Tabula Muris reference. This finding supports an interpretation in which reduced physical function, as represented in the shared factor, is genetically connected to central neural and systemic regulatory contexts in addition to peripheral muscle biology. It also highlights the importance of distinguishing physical-function phenotypes from narrowly defined muscle-cell-specific mechanisms.

Spatial transcriptomic mapping using the E16.5_E1S1.MOSTA neuro-muscle reference provided an additional tissue-contextual layer. The top spatial mapping signals were predominantly annotated as brain, with additional signals in spinal cord regions. This spatial distribution aligns with the pathway and cell-type findings, which highlighted neuronal, synaptic, and glial contexts. Because the reference is derived from mouse embryonic spatial transcriptomic data, the spatial mapping results should be interpreted as localization of genetic signal within a developmental neuro-muscle reference rather than as direct evidence of adult human tissue causality. The convergence of pathway, cell-type, and spatial evidence nevertheless supports a central nervous system-related biological context for the shared factor.

Partitioned heritability analysis added a functional genomic perspective. Stratified LDSC showed that SNP heritability of the shared factor was enriched in conserved sequence annotations as well as intronic, promoter, and chromatin-related annotations. These results suggest that the shared genetic architecture is partly localized to functionally constrained and gene-regulatory genomic regions. This observation is consistent with the TWAS results, because regulatory genomic enrichment provides a plausible genomic context for expression-mediated associations. Technical or genomic-architecture-related annotations, including MAF-adjusted, recombination-related, and background-selection-related features, were treated as covariates rather than primary biological mechanisms.

Taken together, the multilayer evidence supports an exploratory prioritization framework for the genetic overlap between depression-related liability and reduced physical function. The most consistent signals emerged across neuronal and synaptic pathways, brain neuronal and glial cell types, brain and spinal cord spatial contexts, and conserved regulatory genomic annotations. This pattern suggests that central nervous system and gene-regulatory contexts are prioritized within the shared genetic signal, while remaining hypothesis-generating rather than mechanistically conclusive. Endocrine-metabolic and immune-hematopoietic signals may represent additional systemic contexts for future evaluation.

These findings have implications for the genetic interpretation of physical function in the context of depression-related liability. Physical function is often conceptualized through musculoskeletal traits, including strength, lean mass, and mobility. The present results suggest that the genetic overlap between depression-related traits and physical-function-related traits may be substantially influenced by central neural regulation, synaptic organization, and neuroglial biology. This perspective is consistent with clinical observations that depressive symptoms are frequently accompanied by psychomotor slowing, reduced activity, fatigue, altered motivation, and impaired functional performance. Genetic studies of physical decline may therefore benefit from incorporating central nervous system and regulatory genomic frameworks alongside peripheral muscle and metabolic models.

The study also provides a framework for prioritizing candidate genes and biological systems for future work. Convergent genes such as DRD2, TMEM106B, LRFN5, CENPW, NCAPG, DCAF16, SGIP1, and FAM120A can be evaluated in independent datasets, expression resources, and experimental systems. Neuronal and glial cell-type signals provide a rationale for examining brain-region-specific and cell-type-specific expression patterns. The endocrine-metabolic and immune-hematopoietic signals may guide secondary analyses linking shared genetic liability to metabolic biomarkers, inflammatory traits, or systemic aging-related phenotypes. These future directions can help clarify how shared polygenic liability translates into observable patterns of mood-related symptoms and reduced physical performance.

Several limitations should be acknowledged, particularly because this study was designed as a discovery-stage summary-statistics analysis rather than a replication or experimental validation study. First, although Q_SNP analysis was added to evaluate SNP-level heterogeneity, many genome-wide significant signals showed residual heterogeneity, indicating that factor-GWAS loci may reflect a mixture of shared-factor and trait-specific genetic effects. Second, the analysis used GWAS summary statistics rather than individual-level data. The shared factor therefore reflects genetic covariance among proxy phenotypes and cannot directly evaluate within-person clinical co-occurrence, depression duration, or age- and sex-specific effects. Third, the input GWAS datasets were restricted to or predominantly derived from individuals of European ancestry, which may limit generalizability to other populations. Fourth, Genomic SEM models shared genetic covariance but does not determine causal direction among depression-related liability, muscle-related traits, and physical function. Fifth, TWAS identifies associations with genetically predicted expression and remains sensitive to linkage disequilibrium, co-regulation, and model-specific prediction performance [47]; therefore, TWAS- and FOCUS-supported genes should be interpreted as expression-prioritized candidates rather than confirmed causal or colocalized genes, particularly because no formal GWAS-eQTL colocalization, SMR or HEIDI analysis was performed. Sixth, fine-mapping prioritized one high-confidence locus, while other associated regions require larger sample sizes or complementary functional evidence for further resolution. Seventh, CELLECT and gsMap analyses relied on mouse reference resources, including Tabula Muris and embryonic MOSTA spatial transcriptomic data; these findings should be interpreted as contextual localization rather than direct adult human mechanistic validation. Eighth, balance was not available as a separate input GWAS phenotype; therefore, balance-related interpretation was limited to its indirect relevance to walking pace, neuromuscular coordination, and integrated physical performance. Ninth, no independent factor GWAS, non-European ancestry dataset, or experimental validation cohort was available for replication; therefore, the prioritized loci, genes, and biological contexts should be validated in future independent and multi-ancestry studies.

## 5. Conclusions

In conclusion, this study used Genomic SEM to construct a Depression–Physical Function shared genetic factor from two depression-related traits and three physical-function-related traits, and systematically characterized its genetic architecture through multilayer post-GWAS analyses. The shared factor represents a cross-trait genetic covariance dimension and showed stable model fit, measurable SNP heritability, and the expected genetic correlation pattern. Downstream analyses prioritized candidate loci, genes, and biological annotations, with enrichment patterns most consistent with neuronal, synaptic, and regulatory genomic contexts. These findings provide a framework for future validation of shared genetic signals rather than definitive evidence of causal biological mechanisms. Future studies integrating individual-level longitudinal cohorts, multi-ancestry GWAS, tissue-specific expression resources, and experimental validation are warranted to clarify how these shared genetic signals contribute to depression-related functional decline.

## Figures and Tables

**Table 1:** Input GWAS datasets and LDSC-derived SNP heritability estimates. Additional input-trait information, including public data source, GWAS identifier, trait abbreviation, phenotype type, and observed-scale SNP heritability, is provided in Supplementary Table S1.

Domain	Phenotype	GWAS ID	Sample Size	Ancestry	h^2^
Depression-related trait	Depressive symptoms	ukb-a-242	323,267	European	0.0421
Depression-related trait	Depression diagnosis	ebi-a-GCST005902	322,580	European	0.0747
Physical-function-related trait	Grip strength	ebi-a-GCST90014019	406,552	European	0.1198
Physical-function-related trait	Appendicular lean mass	ebi-a-GCST90000025	450,243	European	0.3665
Physical-function-related trait	Walking pace	ukb-b-4711	459,915	European	0.0727

**Table 2:** Genomic SEM model fit and leave-one-trait-out sensitivity analysis.

Model	Phenotype Removed	CFI	SRMR
Full model	None	0.9958	0.0175
Leave-one-trait-out model	Depressive symptoms	0.9971	0.0226
Leave-one-trait-out model	Depression diagnosis	N/A	N/A
Leave-one-trait-out model	Grip strength	0.9956	0.0255
Leave-one-trait-out model	Appendicular lean mass	0.9909	0.0425
Leave-one-trait-out model	Walking pace	1.0000	0.0081

Table note: CFI, comparative fit index; SRMR, standardized root mean square residual. Leave-one-trait-out sensitivity analyses were performed by sequentially removing one constituent phenotype and refitting the Genomic SEM model using the remaining four phenotypes. The model excluding depression diagnosis was saturated; therefore, conventional global fit indices were not applicable. Full model parameters and model-comparison results are provided in Supplementary Tables S3 and S4. N/A, not applicable.

**Table 3:** Genes supported by convergent locus-, gene-, and expression-level evidence. Brief functional annotations for these genes are provided in Supplementary Table S5.

Gene	MAGMA P	TWAS Z	TWAS P	TWAS FDR	Significant TWAS Tissues, n
TMEM106B	1.61 × 10^−11^	7.66	1.80 × 10^−14^	1.96 × 10^−10^	6
CENPW	5.52 × 10^−8^	6.65	2.92 × 10^−11^	6.16 × 10^−8^	12
DRD2	2.47 × 10^−14^	−5.59	2.25 × 10^−8^	2.82 × 10^−5^	6
LRFN5	9.26 × 10^−8^	5.52	3.30 × 10^−8^	4.04 × 10^−5^	1
NCAPG	2.71 × 10^−11^	4.99	6.16 × 10^−7^	2.82 × 10^−4^	10
DCAF16	1.13 × 10^−6^	−4.99	6.16 × 10^−7^	2.82 × 10^−4^	13
SGIP1	1.47 × 10^−8^	4.63	3.66 × 10^−6^	1.23 × 10^−3^	2
FAM120A	2.63 × 10^−7^	−3.96	7.62 × 10^−5^	9.02 × 10^−3^	4

## Data Availability

All GWAS summary statistics used in this study were obtained from publicly available resources. Detailed GWAS identifiers, public data sources, phenotype definitions, sample sizes, ancestry information, phenotype types, and observed-scale SNP heritability estimates are provided in Supplementary Table S1. The post-GWAS summary results generated in this study are provided in the Supplementary Tables; the code is available upon reasonable request; the analyses were conducted using publicly available software and custom scripts. The main software tools and parameters are described in the Section 2. Custom scripts are available from the corresponding author upon reasonable request. Ethics approval and consent to participate were obtained. This study used publicly available summary-level GWAS data and did not involve new individual-level human participant data. Ethical approval and informed consent were obtained in the original studies. No additional ethical approval was required for this secondary analysis.

## Supplementary Materials

The following supporting information can be downloaded at: https://www.mdpi.com/article/10.3390/genes17070813/s1, Table S1: Input GWAS; Table S2: LDSC Diagnostics; Table S3: Genomic SEM; Table S4: Model Comparison; Table S5: Gene Functions.

## Abbreviations

AIC, Akaike information criterion; CFI, comparative fit index; CELLECT, cell-type expression-specific integration for complex traits; FDR, false discovery rate; FUMA, functional mapping and annotation of genome-wide association studies; Genomic SEM, genomic structural equation modeling; GWAS, genome-wide association study; LDSC, linkage disequilibrium score regression; MAGMA, multi-marker analysis of genomic annotation; PIP, posterior inclusion probability; SNP, single-nucleotide polymorphism; SRMR, standardized root mean square residual; TWAS, transcriptome-wide association study.
